# A comparison between the apical and subcostal view for three-dimensional echocardiographic assessment of right ventricular volumes in pediatric patients

**DOI:** 10.3389/fcvm.2023.1137814

**Published:** 2023-05-05

**Authors:** Alessandra M. Ferraro, Kristin Bonello, Lynn A. Sleeper, Minmin Lu, Melinda Shea, Gerald R. Marx, Andrew J. Powell, Tal Geva, David M. Harrild

**Affiliations:** ^1^ Department of Cardiology, Boston Children's Hospital, Boston, MA, United States; ^2^ Department of Pediatrics, Harvard Medical School, Boston, MA, United States; ^3^ PhD Program in Angio-Cardio-Thoracic Pathophysiology and Imaging, Sapienza University of Rome, Rome, Italy

**Keywords:** three-dimensional echocardiography, right ventricular volumes, congenital heart disease, pediatrics, apical view, subcostal view

## Abstract

**Background:**

Accurate measurement of ventricular volumes is an important clinical imaging goal. Three-dimensional echocardiography (3DEcho) is used increasingly as it is more available and less costly than cardiac magnetic resonance (CMR). For the right ventricle (RV), the current practice is to acquire 3DEcho volumes from the apical view. However, in some patients the RV may be better seen from the subcostal view. Therefore, this study compared RV volume measurements from the apical vs. the subcostal view, using CMR as a reference standard.

**Methods:**

Patients <18 years old undergoing a clinical CMR examination were prospectively enrolled. 3DEcho was performed on the day of the CMR. 3DEcho images were acquired with Philips Epic 7 ultrasound system from apical and subcostal views. Offline analysis was performed with TomTec 4DRV Function for 3DEcho images and cvi42 for CMR ones. RV end-diastolic volume and end-systolic volume were collected. Agreement between 3DEcho and CMR was assessed with Bland-Altman analysis and the intraclass correlation coefficient (ICC). Percentage (%) error was calculated using CMR as the reference standard.

**Results:**

Forty-seven patients were included in the analysis (age range 10 months to 16 years). The ICC was moderate to excellent for all volume comparisons to CMR (subcostal vs. CMR: end-diastolic volume 0.93, end-systolic volume 0.81; apical vs. CMR: end-diastolic volume 0.94, end-systolic volume 0.74).The 3DEcho mean % error vs. CMR for end-systolic volume was 25% for subcostal and 31% for apical; for end-diastolic volume it was 15% for subcostal and 16% for apical. The % error was not significantly different between apical vs. subcostal views for end-systolic and end-diastolic volume measurements.

**Conclusions:**

For apical and subcostal views, 3DEcho-derived ventricular volumes agree well with CMR. Neither echo view has a consistently smaller error when compared to CMR volumes. Accordingly, the subcostal view can be used as an alternative to the apical view when acquiring 3DEcho volumes in pediatric patients, particularly when the image quality from this window is superior.

## Introduction

Measures of right ventricular (RV) volumes are critically important from a clinical perspective in the fields of pediatric and adult congenital heart disease (1–7), particularly in conditions providing a volume-loaded RV such as in the setting of an atrial septal defect or dysfunction of the pulmonary or tricuspid valves (7). Accurate measurement of these volumes is essential in the setting of tetralogy of Fallot (ToF), a common condition among pediatric patients (1, 3, 5, 6). In addition, RV volumes, together with RV function, are important parameters in patients status post Fontan palliation due to their correlation with mortality and heart transplant outcomes (8). Similarly, 3DEcho RV volume assessment was able to predict the severity of outcomes in patients with pulmonary hypertension (9). As well, 3DEcho RV volume assessment was the method used to assess differences in RV size and function after either Blalock-Taussig or Sano shunt in a multicenter study (9).

Cardiac magnetic resonance (CMR) is currently considered the reference standard for the measurement of ventricular volumes (10). However, this technology is relatively expensive and time-consuming. As an alternative, three-dimensional echocardiography (3DEcho) for the measurement of ventricular volumes is an emerging technique which has wider availability and lower expense relative to CMR, in addition to the fact that it may be used for patients with a contraindication to CMR. As well, 3DEcho images have a shorter acquisition time and time for analysis (with current semi-automated tools) as little as 3 min (11).

Traditionally, 3DEcho volumes have been acquired from an apical four-chamber view (12–18). However, there are limitations to this view including difficulty visualizing portions of the RV, particularly the outflow, and especially when ventricular dilation is present. These limitations result, in part, from the anterior position of the RV and its location just posterior to the sternum and rib cage (19, 20). Based on these challenges, recent data have called into question the practice of deriving 3DEcho RV volumes images based on apical view (20). An alternative to the apical view is the subcostal view. The potential advantage of this view is access to the entire RV, including the outflow, by avoiding acoustic shadowing from the sternum and rib cage (20, 21). On the other hand, it might happen that patients do not have adequate quality images from this view while having good apical ones. Therefore, in this study we sought to compare 3DEcho RV volume measurements from the apical vs. subcostal view, using contemporaneously acquired CMR measurements as a reference standard.

## Materials and methods

### Study design

Patients who were referred for a clinical CMR were approached prospectively for acquisition of 3DEcho images immediately prior to or following the CMR examination. Inclusion criteria were age ≤18 years old, both a right and left ventricle were present, and adequate apical and subcostal imaging windows. The study protocol was approved by the local Institutional Review Board (IRB-P00033035). Informed consent was obtained from the patient's parent. The 3DEcho was acquired on the same day as the CMR in all cases.

### Image acquisition and analysis

**Figure 1:**
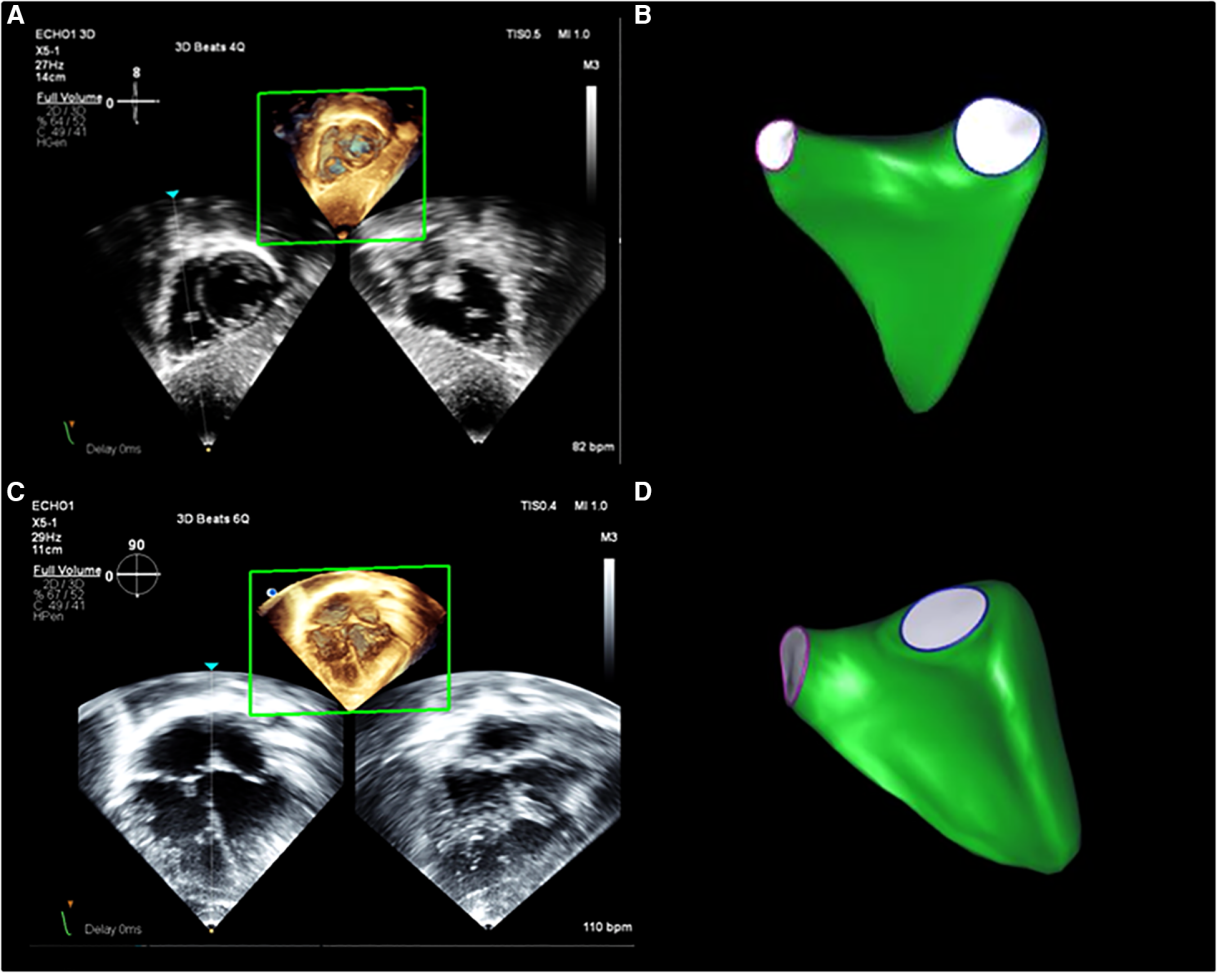
3DEcho RV volume assessment. Example of RV volumes acquired from (**A**) subcostal and (**C**) apical views from a normal patient, as well as examples of the 3D surface resulting from analysis with the post-processing software from subcostal (**B**) and apical (**D**) views.

Echocardiography images were obtained using the Philips EPIQ system (Philips Healthcare, Cambridge, MA, USA). Some patients were sedated for CMR for clinical indications and, in these cases, 3DEcho images were acquired while the patient was recovering from anesthesia; no additional sedation was administered for the 3DEcho images. The X5-1 or X-7-2 transducers were used based upon the patient's size. Images were acquired using standard techniques from subcostal and apical views by sonographers with expertise in 3DEcho (Figure 1). When possible, patients were instructed to hold their breath to minimize “stitch artifact” during imaging reconstruction. A 4 or 6 beat acquisition method was used in all cases.

**Figure 2:**
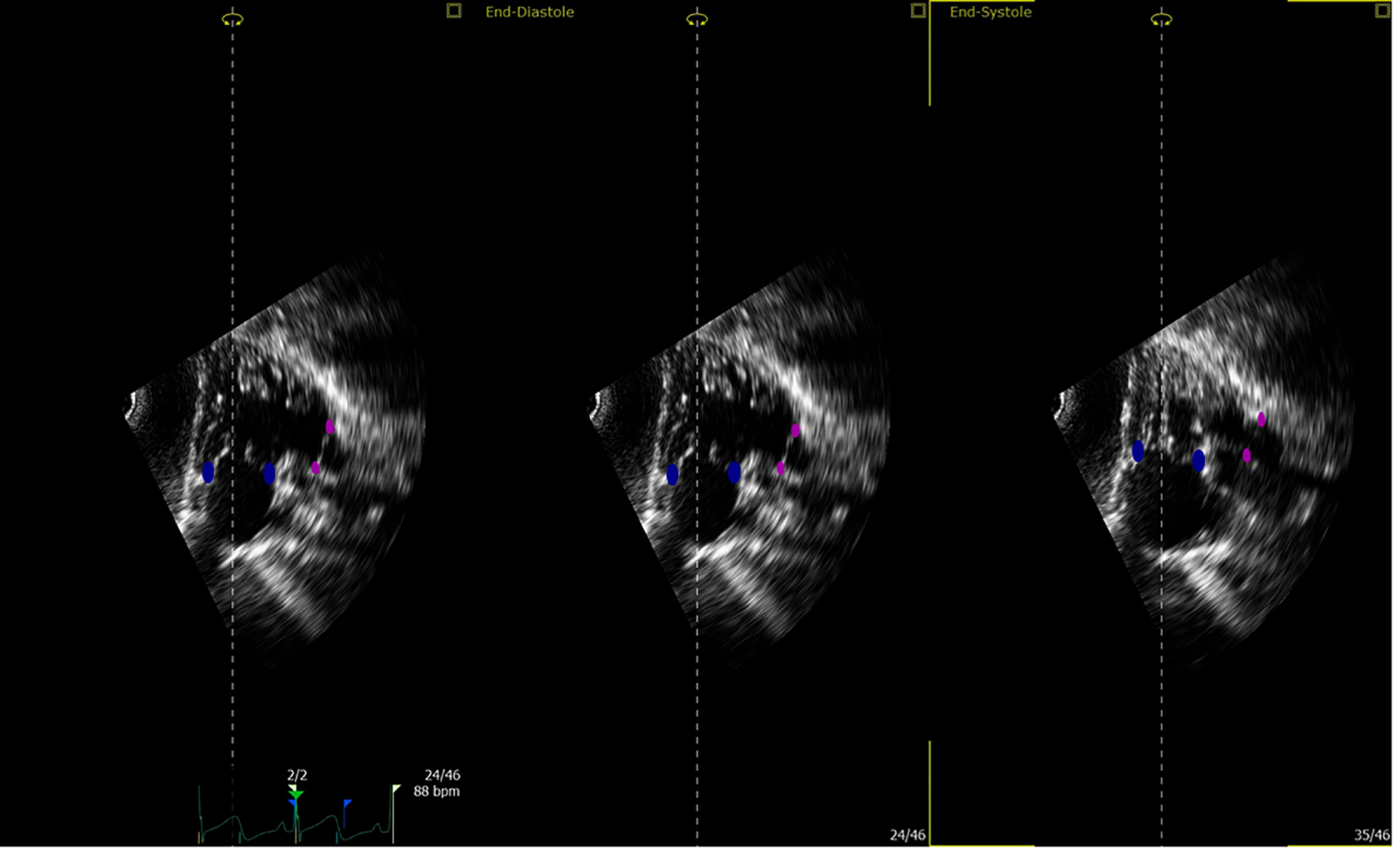
Tricuspid and pulmonary valve landmarks. Blue points define the tricuspid valve annulus; magenta points define the pulmonary valve annulus (this is true for both apical and subcostal views).

**Figure 3:**
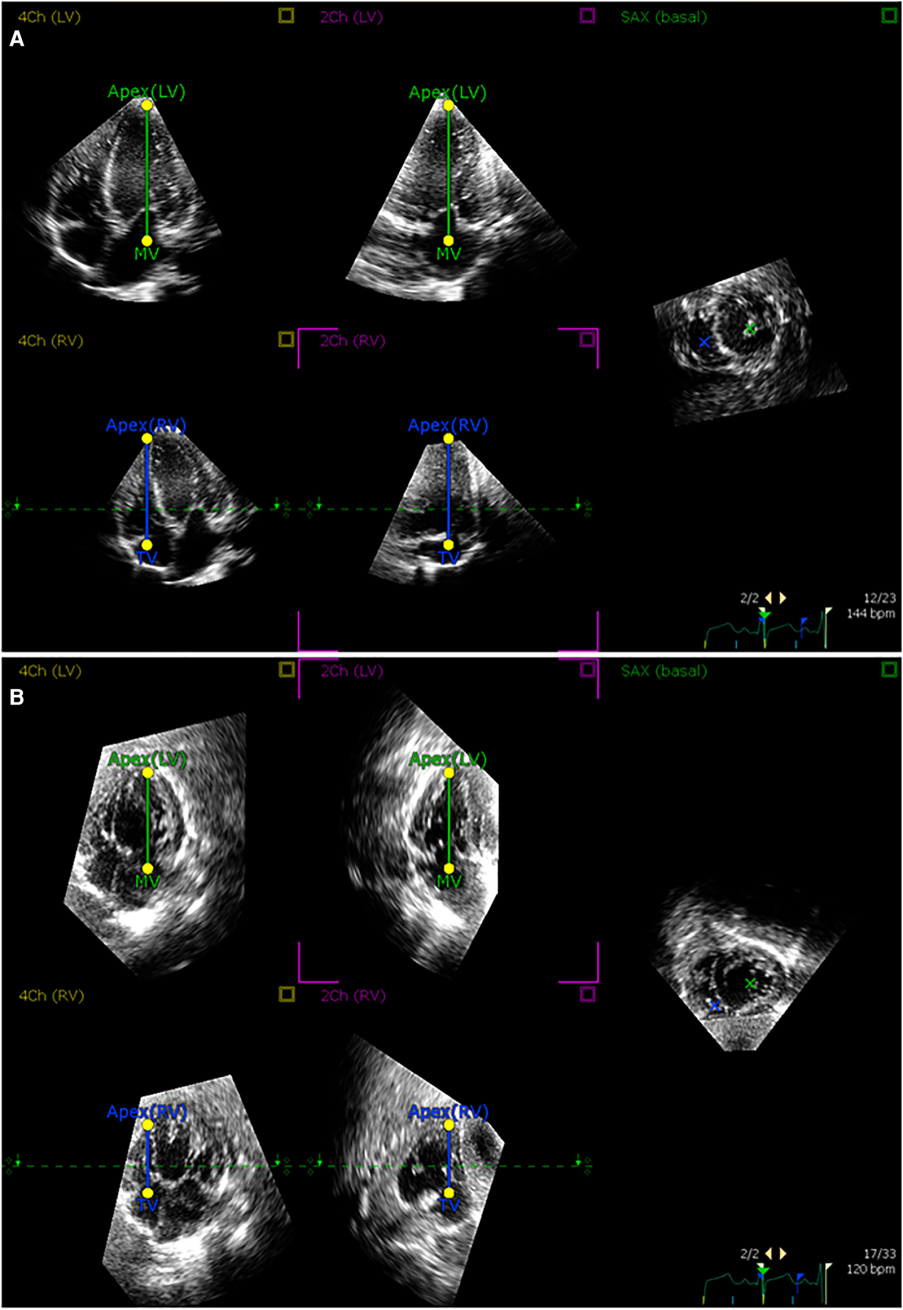
Alignment of 3D echocardiographic images from apical and subcostal views. An example of alignment of (**A**) apical and (**B**) subcostal views in the postprocessing software according to prespecified landmarks.

Deidentified images were stored in Digital Imaging and Communications in Medicine format. Offline volumetric analysis was carried out with 4D RV Function version 3 (TomTec, Unterschleißheim, Germany) according to the manufacturer's recommendations and prior descriptions (22). Manual adjustments of the endocardial borders as well as the tricuspid and pulmonary valves landmarks were made following the generation of the semi-automatic tracing (Figure 2). Custom bookmark tools were constructed to optimize the consistency of image alignment acquired from the subcostal view (Figure 3). End-diastolic volume (EDV) and end-systolic volume (ESV) were recorded. Intra- and inter-observer reproducibility was assessed for 10 randomly selected patients. For inter-observer measurements, the second analysis was performed at least 2 weeks after the first.

All CMR images were obtained from a 1.5 Tesla CMR scanner (Achieva, Phillips Healthcare, Best, the Netherlands). Imaging included a 12–14 slice stack (slice thickness 8–10 mm) of breath hold, ECG-gated, balanced steady-state free precession (bSSFP) cine acquisitions in the short-axis plane. Ventricular volumes were measured using commercially available software (cvi42, Circle Cardiovascular Imaging Inc., Calgary, Alberta, Canada; and QMass, Medis Medical Imaging Systems, Leiden, the Netherlands). Horizontal (four-chamber) and vertical (two-chamber) long-axis images were used as cross-references to aid with the identification of the ventricular myocardium to be included as chamber volume. The left ventricular papillary muscles and major trabeculations of the RV (e.g., septal band) were excluded from the blood pool and considered part of the myocardial mass as previously described (1, 2, 3).

### Statistical analysis

Agreement between 3DEcho and CMR was assessed with Bland-Altman analysis and the intraclass correlation coefficient (ICC). To study if age may have an impact on agreement, we additionally divided our cohort in three age groups: <6 years old, 6–2 years old, and >12 years old. Percentage (%) error was calculated as [|(Echo − CMR)|/mean of Echo and CMR] × 100. Differences in raw values and % error for apical vs. subcostal views were compared with a paired *t*-test. In addition, differences in cardiac output using ventricular volumes calculated with apical vs. subcostal views were assessed using a Wilcoxon signed rank exact test.

Intra- and inter-observer reproducibility was assessed with a one-sample *t*-test and ICC. The Bland-Altman plots were used to display agreement between two readings (from the same observer) and between two readings (from different observers). Descriptive statistics include mean ± standard deviation and median with interquartile range. A *p* value 0.05 was considered to be statistically significant.

## Results

### Study participants

Fifty patients were consented for the study; in 3 of these, however, 3DEcho image quality from one of the views was judged to be inadequate for analysis upon subsequent review (apical view 1, subcostal view 2). Hence, the analytic cohort size for apical vs. subcostal comparisons was 47 patients. Demographic and clinical characteristics are presented in Table 1. Ages ranged from 10 months to 16 years. Eighteen patients underwent general anesthesia for CMR. Indications for CMR were for suspected or established congenital heart disease in nearly all patients (*n *= 43).

**Table 1:** Patient demographics (*n* = 47).

Characteristics	Value
**Age (years)** (median and range)	9.5 (0.8–16)
Female (%)	29% (14)
Body surface area (m^2^)	1 (0.7–1.3)
Body mass index (kg/m^2^)	16.5 (15.5–19.2)
Height (cm)	131.0 (109.3–149.0)
Heart rate (bpm)	80 (73.0–96.0)
**CMR**	
RV EDV, ml	92.7 (67.5–123.7)
RV ESV, ml	42.7 (26.7–56.7)
**Diagnosis**	
Aortic stenosis	6
Atrioventricular canal defect	4
Atrial septal defect	3
Ventricular septal defect	3
Shone syndrome	3
Double outlet right ventricle	2
Partial anomalous pulmonary venous return	2
Coarctation of the aorta	2
Tetralogy of Fallot	2
Other congenital heart disease	10
Other non-congenital heart disease	10

Data are presented as median and interquartile ranges (except for age). EDV, end-diastolic volume; ESV, end-systolic volume. Other congenital heart disease includes: dysplastic tricuspid valve; double chamber right ventricle; hypoplastic left heart syndrome; left ventricular non-compaction; mitral valve prolapse; pulmonary valve stenosis and atresia; transposition of the great arteries. Other non-congenital heart disease includes: alpha and beta thalassemia; hepatoblastoma; lymphatic malformation; Loeys-Dietz syndrome; multi-inflammatory disease syndrome in children; pulmonary hypertension; sickle cell disease; ventricular ectopy. Two patients were judged to have normal cardiac structure and function.

### Comparison of volume measurements by 3DEcho apical and subcostal views to CMR

**Figure 4:**
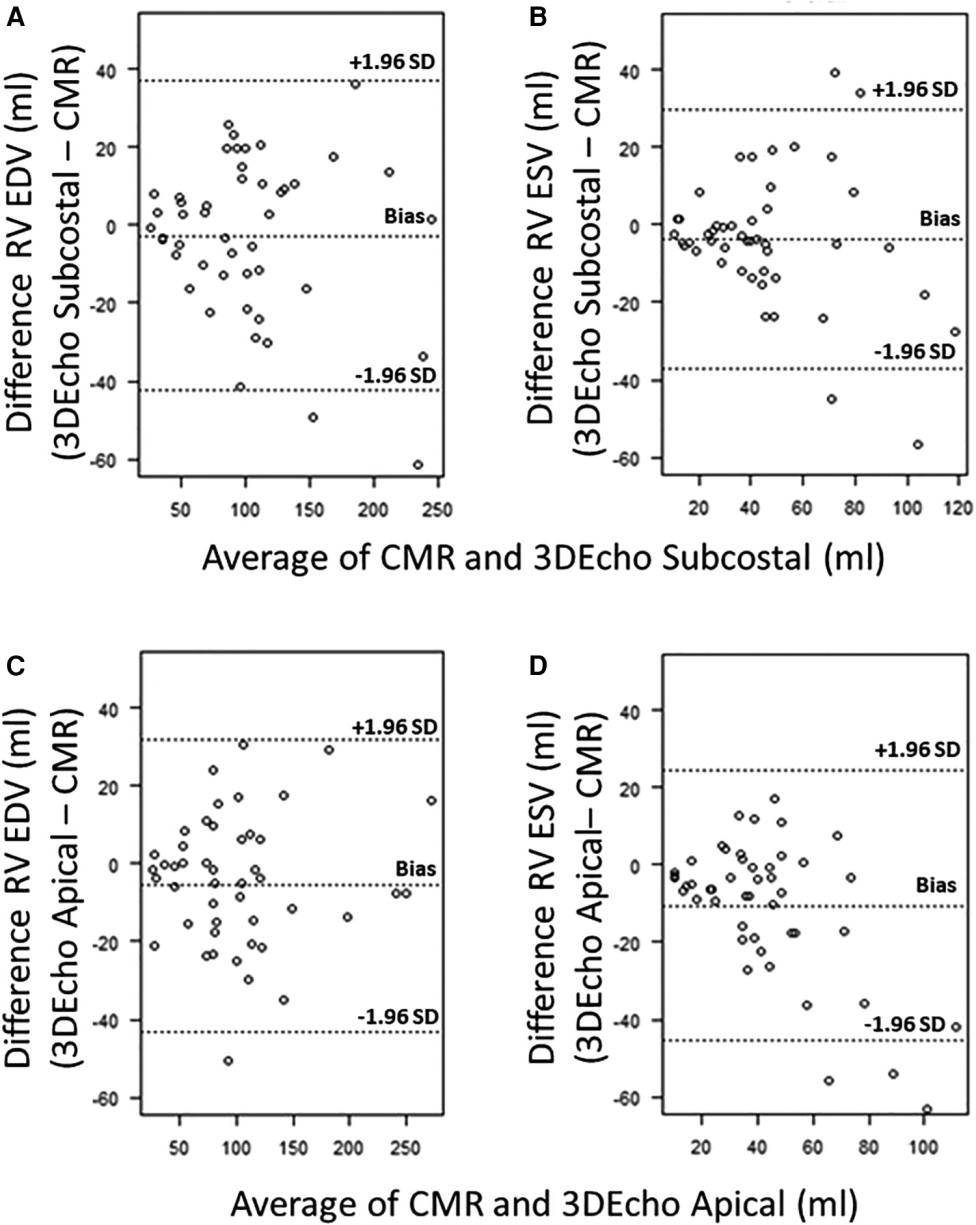
Bland–Altman plots for 3DEcho vs. CMR volumetric measurements (*N* = 47). Bland–Altman plots for 3DEcho vs. CMR for: end-diastolic measurements (panels **A,C**); end-systolic measurements (panels **B,D**). CMR, cardiac magnetic resonance; EDV, end diastolic volume; ESV, end systolic volume; ml, milliliter; RV, right ventricular.

**Figure 5:**
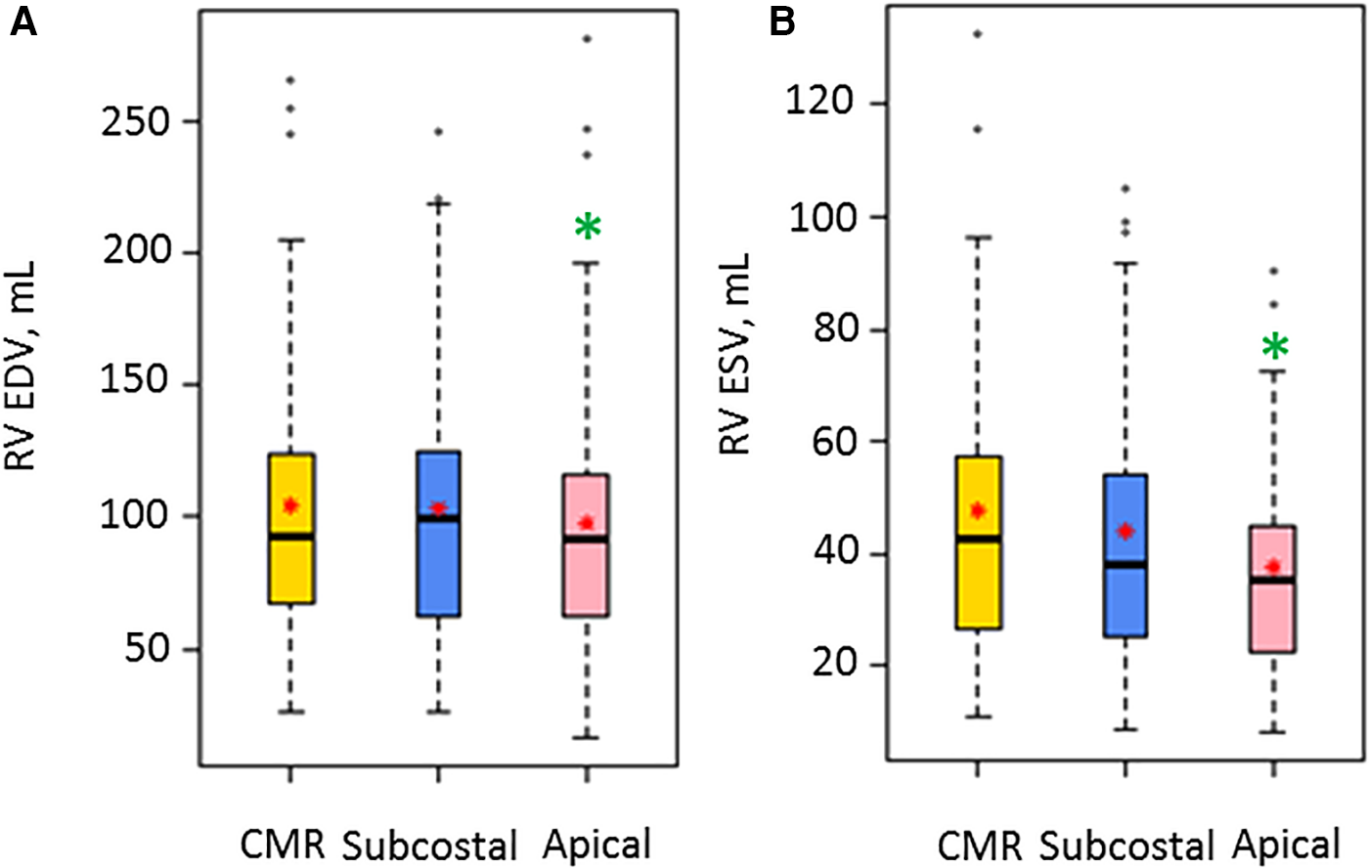
Box plots for volume measurements by CMR and the two 3D Echo views (*N* = 47). EDV, end diastolic volume; ESV, end systolic volume; ml, milliliter; RV, right ventricular. A red star indicates the mean value, and a solid box line indicates the median value. A green asterisk shows significant differences for the 3DEcho view relative to CMR.

Bland-Altman plots for 3DEcho vs. CMR measurements of ventricular volumes for the two echo views are presented in Figure 4. In addition, differences in cardiac output using ventricular volumes calculated with apical vs. subcostal views were assessed using a Wilcoxon signed rank exact test. The biases were not significantly different for the comparison of end-diastolic measurements (panel A vs. panel C) (*p* value = 0.36), but they were different for end-systolic (panel B vs. panel D) (*p*-value <0.05). The biases were negative for all volume comparisons, reflecting an underestimation of 3DEcho ventricular volumes compared to CMR. Figure 5 presents box plots for the volume data analyzed in a grouped fashion. Volumes measured from the apical windows were significantly smaller than the CMR values (*p* values for EDV = 0.05 and ESV <0.001); the volumes measured by subcostal windows and the CMR values were not statistically different. When comparing mean volumes from subcostal to apical view: EDV volume from apical (98.5 ml) and subcostal (101.5 ml) views did not differ from each other, *p* = 0.36; ESV volume from apical (37.0 ml) and subcostal (43.9 ml) views differed one from the other (*p* < 0.05). Table 2 presents mean difference data between the two views compared to CMR, both as a raw value and as a percent error. The % error was not statistically different between apical vs. subcostal views for ESV and EDV measurements. Agreement as assessed by the ICC between 3DEcho and CMR for each view was high (ICC >0.7 for all), and higher for EDV compared to ESV (Table 2). When stratified by age, the patients in the middle age group (6 to 12 years) had a lower ICC than patients in both the youngest (<6 years) and oldest (>12 years) age groups. A factor contributing to these differences may be variations in patient diagnoses among the three patient groups; for example, the 6–12 year old cohort had a higher prevalence of patients with small left-sided structures than the other two.

**Table 2:** Comparisons of RV volumes calculated by 3DEcho views vs. CMR; mean values, absolute percent error, and ICC.

	Mean difference	Absolute %error	ICC			
	All		All	<6 years	6–12 years	>12 years
	(*n* = 47)			*n* = 14	*n* = 21	*n* = 12
**Subcostal vs. CMR**						
EDV, ml	−2.8 ± 20.2	15.2 ± 9.4	0.93	0.92	0.61	0.89
ESV, ml	−3.7 ± 17.1	25.0 ± 16.7	0.81	0.91	0.36	0.79
**Apical vs. CMR**						
EDV, ml	−5.7 ± 19.1	15.6 ± 15.3	0.94	0.82	0.61	0.95
ESV, ml	−10.6 ± 17.76	30.7 ± 21.1	0.74	0.67	0.34	0.61

CMR, cardiac magnetic resonance; EDV, end-diastolic volume; ESV, end-systolic volume; ICC, interclass correlation coefficient.

The median and interquartile range for cardiac output were: subcostal view, 4,312 (2,893–5,711) ml/min; apical view, 4,296 (2,802–6,078) ml/min; these did not differ statistically (*p* = 0.42).

### Assessment of 3DEcho volume measurement reproducibility

Inter- and intra-observer reproducibility data for RV EDV and RV ESV volume measurements using the two views are presented in Table 3. ICC values are in the good to excellent range (all >0.75) with slightly higher values for all EDV volumes measurements compared to ESV. The mean difference between the two measurements was statistically significant only for inter-observer subcostal RV ESV (*p* = 0.03).

**Table 3:** Intra and inter-observer reproducibility for 3D Echo according to view (*n* = 10).

	Mean difference O2–O1 95% CI	Paired test *p*	Absolute % error O2–O1	ICC (95%CI)
**Intra-observer**				
**Subcostal**				
RV EDV	−2.5 ± 15.2 (−32.4 to 27.3)	0.61	13.8 ± 9.6	0.96 (0.94, 0.98)
RV ESV	2.9 ± 10.4 (−17.5 to 23.2)	0.41	17.2 ± 16.6	0.89 (0.82, 0.94)
**Apical**				
RV EDV	3.8 ± 17.8 (−31.2 to 38.7)	0.52	13.8 ± 14.4	0.96 (0.93, 0.98)
RV ESV	6.5 ± 9.7 (−12.5 to 25.5)	0.06	23.9 ± 16.7	0.90 (0.84, 0.94)
**Inter-observer**				
**Subcostal**				
RV EDV	9.49 ± 26.25 (−41.96, 60.94)	0.28	20.8 ± 21.0	0.88 (0.80, 0.93)
RV ESV	13.11 ± 15.90 (−18.05, 44.26)	**0**.**03**	36.2 ± 18.8	0.76 (0.61, 0.85)
**Apical**				
RV EDV	8.73 ± 23.65 (−37.62, 55.09)	0.27	18.8 ± 15.6	0.92 (0.87, 0.96)
RV ESV	8.11 ± 13.28 (−17.91, 34.13)	0.09	33.4 ± 17.9	0.82 (0.70, 0.89)

EDV, end diastolic volume; ESV, end systolic volume; O1 and O2, observer 1 and 2; RV, right ventricular.

## Discussion

In this study, we compared 3DEcho RV volume measurements from subcostal vs. apical views using CMR as the reference standard in a pediatric population. Overall, the volumes derived from echocardiography agreed well with those from CMR, for both systolic and diastolic measurements. In addition, we found no significant difference in the percent error of the measured volumes between the two views relative to CMR, thus, neither view clearly emerged as consistently inferior relative to the other.

### Apical and subcostal view

The great majority of descriptions of 3DEcho RV volume acquisitions have used the apical view in a wide range of ages, in both normal and abnormal hearts (13–15, 20, 22, 24–26). In the pediatric population, the feasibility of quantifying 3D RV volumes from this view has varied widely with reported ranges between 20% (20) to 91% (14). 3DEcho RV volumes acquired from the apical view have been reported to correlate well with CMR (14, 22); however, there are limitations to acquisition from this view. For example, when the RV is dilated, portions of the ventricle may be incompletely captured from apical imaging, particularly the region of the heart adjacent to the transducer and the right ventricular outflow (14). In these cases, the 3D RV volumes may be significantly underestimated (13) (for example, inconsistent representation of the outflow tract). To overcome this issue, the subcostal view has been proposed as an alternative view for acquiring 3D RV volumes (20). In one early report describing its use in a pediatric cohort, the authors describe that analysis was feasible in 44% of the patients using this view, with only 20% feasibility in the same patients using the apical window. In our study, the feasibility of analysis was not examined; nearly all images could be analyzed as subjects had been preselected for having good-quality images from both views.

### Comparison of 3D RV volumes to CMR

Comparisons of 3DEcho-based RV to CMR have been reported in multiple studies and nearly all have used the apical view for the echo-based quantification. For example, Dragulescu et al. compared 3DEcho RV volume images to CMR in 36 pediatric patients ages 7–18 years. They found that 3D RV EDV and ESV correlated very well with CMR (correlation coefficients were 0.99 for both EDV and ESV). In their report, Dragulescu et al. highlighted the importance of manually adjusting the endocardial borders and landmarks (such as tricuspid and pulmonary valves) to increase correlation of 3DEcho (14); this was similar to the techniques which we used in the current study. In a later report, Laser et al. (22) also found that 3DEcho RV volumes (EDV, ESV) were highly correlated to CMR (*r* values 0.98 for both volumes). Similarly, Muraru et al., in a cohort of congenital heart disease patients that included both pediatric and adult patients, showed that 3DEcho RV volumes correlated highly with CMR values (*r* = 0.92 and 0.93 for ESV and EDV, respectively) (11). Similar to these descriptions, our EDV measurements, from the apical view, agreed well with the CMR (ICC 0.94). ESV measurements, however, were less reliable (ICC 0.74). Some of the difference in our findings and those reported in the literature might be explained by differences in patient size and age, and the nature of the congenital heart disease in the included cohort, with variations in the degree of dilation of the portions of the RV that are particularly difficult to image by 3DEcho.

To the best of our knowledge, the only other study that has compared 3DEcho RV volumes acquired from the subcostal view to CMR measurements was also from our group (27). In this report, we studied a pediatric population ages 2 to 8 years with a variety of forms of single ventricle congenital heart disease. EDV and ESV agreed well with CMR values (ICCs 0.95 and 0.94, respectively) (27). In the current study, 3DEcho-based EDV from the subcostal view also agreed well with CMR (ICC = 0.93). ESV agreement was good (ICC 0.81) but somewhat less robust. In a subgroup analysis, agreement was best in the youngest cohort, perhaps reflecting enhanced image quality in this group of patients relative to the older cohort.

### Reproducibility

Prior reports of RV volume measurements (both EDV and ESV) have described good to excellent intra- and inter-observer reproducibility with less reproducibility for intra-observer readings (14, 17, 18, 20, 27, 28). Our results for intra- and intra-observer reproducibility follow a similar pattern with ICC values reflecting good agreement. Values were generally higher for intra-observer measurements, compared to inter-observer, as is typically the case, and were higher for end-diastolic volume compared to end-systolic volume. The latter finding is likely a reflection of the highly trabeculated nature of the RV leading to difficulty identifying the optimal location for placing the end-systolic contour.

### Study limitations

While CMR was considered a reference standard, this technology itself has a certain amount of intrinsic variability in volume measurement. However, CMR is considered the most reliable imaging modality for the assessment of ventricular volumes. Our patients did not have the echo and CMR performed at precisely the same time. However, 3DEcho was performed soon before or after the CMR to mitigate change in patient conditions as much as possible. Finally, while differences are reported as “statistically significant”, these differences may not necessarily have clinical importance.

## Conclusions

3DEcho measurements of RV volumes based on either subcostal or apical views agree well with corresponding values from CMR. There were no significant differences in the errors calculated by either view relative to CMR. These results support the use of the subcostal view as an alternative to the apical view when acquiring 3DEcho volumes in pediatric patients with good subcostal views, particularly if the RV outflow is not well seen in the apical view. These findings may encourage the use of 3DEcho measurement of RV volumes in the pediatric population, particularly when access to CMR is limited.

## Data Availability

The raw data supporting the conclusions of this article will be made available by the authors, without undue reservation. The data and materials from this study will be available upon request and following approval from our Institutional Review Board.
